# Case Report: A *BRCA2* Mutation Identified Through Next-Generation Sequencing in a Birt–Hogg–Dubè Syndrome Family

**DOI:** 10.3389/fonc.2022.835346

**Published:** 2022-02-14

**Authors:** Erika Bandini, Ilaria Cangini, Valentina Arcangeli, Mila Ravegnani, Virginia Andreotti, Giovanna Prisinzano, Lorenza Pastorino, Giovanni Martinelli, Fabio Falcini, Daniele Calistri, Valentina Zampiga, Rita Danesi

**Affiliations:** ^1^ Biosciences Laboratory, IRCCS Istituto Romagnolo per lo Studio dei Tumori (IRST) “Dino Amadori”, Meldola, Italy; ^2^ Romagna Cancer Registry, IRCCS Istituto Romagnolo per lo Studio dei Tumori (IRST) “Dino Amadori”, Meldola, Italy; ^3^ IRCCS Ospedale Policlinico San Martino, Genetics of Rare Cancers, Genoa, Italy; ^4^ Genetics of Rare Cancers, Department of Internal Medicine and Medical Specialties, University of Genoa, Genoa, Italy; ^5^ Scientific Directorate, IRCCS Istituto Romagnolo per lo Studio dei Tumori (IRST) “Dino Amadori”, Meldola, Italy

**Keywords:** case report, Birt–Hogg–Dubé, FLCN, BRCA2, NGS

## Abstract

Birt–Hogg–Dubé syndrome (BHDS) is a rare autosomal dominant inherited disorder caused by a mutation in folliculin (*FLCN*) gene transmitted *via* germline autosomal dominant pattern. Patients with this syndrome have an increased susceptibility to renal cell carcinoma, lung cysts, spontaneous pneumothorax, and benign skin hamartomas, and its diagnosis is not easy and consequently underestimated. Several mutations have been identified in *FLCN* gene, among which the majority of alterations are frameshift (insertion/deletion), nonsense, or splice-site mutations that generally produce unfunctional truncated FLCN proteins. Our aim is to present a case of a BHDS family whose proband is a 56-year-old patient who has been experiencing multiple disorders, has an *FLCN* genetic mutation, and has also been identified to have a pathogenic variant in *BRCA2* gene. Our further purpose is to emphasize the importance of the next-generation sequencing (NGS) approach to identify potential multiple germline mutations in complex and rare oncologic disorders, allowing strict and more targeted cancer screening programs.

## Introduction

Birt–Hogg–Dubé syndrome (BHDS) is a rare autosomal dominant inherited disorder described firstly in 1977, due to the germline transmission *via* an autosomal dominant pattern of a mutation in folliculin (*FLCN*) gene (1). The syndrome is considered to be underdiagnosed due to variable and atypical symptoms, and disease severity can differ significantly even within the same family. Its clinical manifestation is generally associated with multiple pulmonary cysts, frequent spontaneous pneumothoraces (PNXs), benign skin hamartomas, cutaneous fibrofolliculomas, and renal tumors of different histological types (2). *FLCN*, located on chromosome 17p11.2, is currently the main gene known to be associated with BHDS (3). A broad range of mutations have been identified in *FLCN* gene, among which the majority of *FLCN* alterations identified in the germline of BHD patients are frameshift (insertion/deletion), nonsense, or splice-site mutations that generally produce unfunctional truncated FLCN proteins. The most frequently observed mutation is the c.1285dupC/delC, an insertion/deletion of a cytosine in a C8 tract in exon 11 (4, 5). Early and precise diagnosis of BHDS is crucial for clinicians, firstly to better distinguish BHDS patients from others affected by other diseases and secondly to guarantee accurate counseling and screening programs and to ensure targeted treatments, especially in renal cancer patients and their family members. Herein, we present a BHDS family whose proband is a 56-year-old woman suspected to be affected by BHDS and whose diagnosis was confirmed by the mutation of *FLCN* gene. Furthermore, concomitant with the c.1285dupC; p.His429Profs*27 frameshift mutation in *FLCN* gene, we reported, as an incidental finding, the pathogenic variant c.7180A>T; p.(Arg2394Ter) in *BRCA2* gene that was further observed in some family members.

## Case Presentation

A 56-year-old woman, born from non-consanguineous parents, was recruited by our Oncogenetic Counselling Unit because of her personal multiple tumor history: two melanomas, thyroid, parathyroid, and bilateral kidney cancers. She had four admissions to the hospital for acute dyspnea episodes associated with spontaneous PNX that has been treated with talc, pleurodesis, and apicoectomy. Clinical dermatological examination reported the presence of freckles and the absence of cutaneous fibrofolliculomas and trichodiscomas or other skin lesions.

She admitted frequent sunburn during childhood, adolescence, and adulthood. She never made lamps, neither before nor after she was 35 years old. Currently, she does not smoke and does not drink alcohol. She had never used oral contraceptives or hormone replacement therapy. She pointed out having allergies to Augmentin (amoxicillin and clavulanic acid), Valium (diazepam), and Contramal (tramadol). There was no known family history of PNXs, dermatological lesions, or renal cell cancer (RCC). Prior to the PNX episode, she experienced multiple tumors at different sites, firstly developing two melanomas followed by thyroid, parathyroid, and bilateral kidney cancers, despite no previous family cases. Due to the nature of these disorders, she was suspected to be suffering from BHDS according to the diagnostic criteria proposed by European Birt–Hogg–Dubé Consortium (**Table 1**) (6), and a genetic test was required and performed by our Genetics Unit, confirming the diagnosis. After confirmation of the *FLCN* pathogenic variant causing the syndrome, the patient further developed multiple disorders, including kidney renal papillary cell carcinoma, liver metastasis, and metastatic bilateral renal cell carcinoma and she underwent some interventions, as summarized in **Table 2**. She was recently diagnosed with stage 1 breast invasive ductal carcinoma (IDC) according to the American Joint Committee on Cancer [AJCC] Cancer Staging Manual (7): the tumor was characterized by neoplastic cells positive for the expression of estrogen receptors (80%), progesterone receptors (30%), and Ki67 (5%) and negative for the expression of *HER2*/neu. Since she is firstly on dialysis and undergoing chemotherapy following secondary liver disorders, she cannot be subjected to surgery. In consideration of the size of the lesion, she started aromatase inhibitor (AI) therapy with letrozole. At present, the patient has been in a stable condition with tolerable life quality and is still in continuous dialysis treatment and nivolumab administration at a 240-mg dose for kidney metastases, although she is still not suitable for operation.

**Table 1:** Diagnosis criteria for BHD syndrome proposed by the European Birt–Hogg–Dubé Consortium.

Major criteria	Minor criteria
● At least 5 fibrofolliculomas or trichodiscomas, at least 1 histologically confirmed, of adult onset	● Multiple lung cysts: bilateral basally located lung cysts with no other apparent cause, with or without spontaneous primary pneumothorax
● Pathogenic *FLCN* germline mutation	● Renal cancer: early-onset (<50 years) or multifocal or bilateral renal cancer, or renal cancer of mixed chromophobe and oncocytic histology
	● A first-degree relative with BHD

BHD, Birt–Hogg–Dubé.

**Table 2:** Clinical history of proband patient.

Age at diagnosis	Developed pathology and intervention
36	Back melanoma removal
38	Kidney renal clear cell carcinoma (KIRC) and left nephrectomy
39	Melanoma of the right leg removal
41, 46	Multiple left and right renal oncocytomas and cryoablation
50	Left parotid oncocytoma and parotidectomy
50	Papillary thyroid carcinoma (PTC) and thyroidectomy, treatment with 1,850 MBq of I-131
50	Lymph node metastases
51	Kidney renal papillary cell carcinoma type I (KIRP)
51	Breast intraductal papilloma; quadrantectomy of the breast
52	*BRCA* and *FLCN* mutations (BHD)
53	Kidney renal papillary cell carcinoma type II (KIRP) and right radical nephrectomy
54	Liver metastasis from kidney carcinoma
55	Bilateral nephrectomy for metastatic bilateral renal cell carcinoma; treatment with sunitinib
56	Hepatic secondary disorders
57	Therapy with nivolumab; breast infiltrating ductal carcinoma, dialysis, and treatment with letrozole
57	Melanoma enlargement removal

BHD, Birt–Hogg–Dubé.

## Materials and Methods

### Patient Sample Collection

Patients with a history of BHD and referred for genetic counseling of the Genetics Unit of IRST IRCCS were included in the study and enrolled between 2015 and 2020. The study was approved by the institutional review board (Ethics Committee IRST IRCCS-AVR, 2207/2012) and conducted in accordance with the Declaration of Helsinki. Written informed consent was obtained from all subjects before the study. Information about possible tumors and malignancies related to family history of first- and second-degree relatives was also collected.

### Blood Collection and DNA Extraction

Peripheral blood samples were collected and stored at −80°C at the Biosciences Laboratory of the IRCCS Istituto Romagnolo per lo Studio dei Tumori “Dino Amadori.” Genomic DNA was extracted using the QIAamp DNA mini Kit (Qiagen, Hilden, Germany) according to the manufacturer’s instructions. DNA was quantified by Qubit fluorometer (Thermo Fisher Scientific, Waltham, MA, USA) with Qubit dsDNA BR Assay Kit (Thermo Fisher Scientific, Waltham, MA, USA).

### Next-Generation Sequencing

Two panels containing targeted genes, *FLCN* and *BRCA2*, were used for next-generation sequencing (NGS). Genetic analysis of the proband and a part of the relatives was performed using the enrichment protocol TruSight Cancer (Illumina, San Diego, CA, USA), which is an enrichment protocol for the simultaneous sequencing of 94 genes involved in the main hereditary cancer syndromes, starting at 50 ng of genomic DNA to create sequencing libraries (**Table 3**). The panel covers a total of 355 kb and includes the entire coding regions of the 94 genes and the flanking introns (50 bp upstream and downstream of each exon). Raw de-multiplexed reads from MiSeq sequencer were aligned to the reference human genome (UCSC-Build37/hg19) using the Burrows–Wheeler algorithm, running in paired-end mode. To ensure good call quality and to reduce the number of false positives, samples underwent Base Quality Score Recalibration (BQSR), through the Genome Analysis Toolkit GATK, version 3.2.2. After BQSR, sequences around regions with insertions and deletions (indels) were realigned locally with GATK. The MarkDuplicates tool was used to remove duplicate read-pairs that have arisen as artifacts during either PCR amplification or sequencing. For variant analysis, UnifiedGenotyper of GATK was used to search for single-nucleotide variants (SNVs) and indel. Genomic and functional annotations of detected variants were made by Annovar. Coverage statistics was performed by DepthOfCoverage utility of GATK. BASH and R custom scripts were used to obtain the list of low coverage (50×) regions per sample. The regions under this threshold were considered not evaluable. The potential impact of amino acid changes (MAPP p-value) was assessed with PolyPhen-2 HVAR and SIFT. A further part of the subjects was analyzed using the enrichment protocol of SOPHiA Hereditary Cancer Solution (HCS) v1.1 by SOPHiA GENETICS (Saint-Sulpice, Switzerland), which investigates 26 cancer predisposition genes (*ABRAXAS1*, *APC*, *ATM*, *BARD1*, *BRCA1*, *BRCA2*, *BRIP1*, *CDH1*, *CHEK2*, *EPCAM*, *MLH1*, *MRE11*, *MSH2*, *MSH6*, *MUTYH*, *NBN*, *PALB2*, *PIK3CA*, *PMS2*, *PTEN*, *RAD50*, *RAD51C*, *RAD51D*, *STK11*, *TP53*, and *XRCC2*) and pseudogene *PMS2CL* (**Table 4**). Sequencing libraries were created from 200 ng of genomic DNA. Sequences were mapped to the human reference genome GRCh37/hg19. Data output files (FASTQ) were uploaded on the SOPHiA DDM Platform v5.5.0 (SOPHiA GENETICS, Saint-Sulpice, Switzerland) for analysis. The multigene panel (MGP) testing targets a total of 105 kb of the human genome and their flanking regions (on average 25 bp upstream and downstream of each exon). The sequencing was performed using the MiSeq sequencer platform (Illumina) and MiSeq Reagent Kit v2 or MiSeq Reagent Kit v3 600 cycles, configured 2 × 151 cycles in accordance with the manufacturer’s instructions. The bioinformatics analysis of NGS results was performed through Miseq Reporter software (Illumina) and processed with a customized pipeline for TruSight analysis. Furthermore, to validate the FLCN variant c.1285dupC; p.(H429fs), specific intronic primer pairs of exon 11 of FLCN (LRG_325, NG_008001, NM_144997.5) were designed, using the Primer3 algorithm (https://primer3plus.com), a primer designing tool. Purified PCR products were sequenced, with the same primer of the PCR amplification, using the BigDye Terminator v1.1 cycle sequencing kit (Life Technologies, Carlsbad, CA, USA) and a 3130xL Genetic Analyzer (Life Technologies) according to the manufacturer’s instructions.

**Table 3:** Panel of 94 predisposition genes used for NGS analysis through TruSight Cancer.

*AIP*	*ALK*	*APC*	*ATM*	*BAP1*	*BLM*	*BMPR1A*	*BRCA1*	*BRCA2*	*BRIP1*
*BUB1B*	*CDC73*	*CDH1*	*CDK4*	*CDKN1C*	*CDKN2A*	*CEBPA*	*CEP57*	*CHEK2*	*CYLD*
*DDB2*	*DICER1*	*DIS3L2*	*EGFR*	*EPCAM*	*ERCC2*	*ERCC3*	*ERCC4*	*ERCC5*	*EXT1*
*EXT2*	*EZH2*	*FANCA*	*FANCB*	*FANCC*	*FANCD2*	*FANCE*	*FANCF*	*FANCG*	*FANCI*
*FANCL*	*FANCM*	*FH*	*FLCN*	*GATA2*	*GPC3*	*HNF1A*	*HRAS*	*KIT*	*MAX*
*MEN1*	*MET*	*MLH1*	*MSH2*	*MSH6*	*MUTYH*	*NBN*	*NF1*	*NF2*	*NSD1*
*PALB2*	*PHOX2B*	*PMS1*	*PMS2*	*PRF1*	*PRKAR1A*	*PTCH1*	*PTEN*	*RAD51C*	*RAD51D*
*RB1*	*RECQL4*	*RET*	*RHBDF2*	*RUNX1*	*SBDS*	*SDHAF2*	*SDHB*	*SDHC*	*SDHD*
*SLX4*	*SMAD4*	*SMARCB1*	*STK11*	*SUFU*	*TMEM127*	*TP53*	*TSC1*	*TSC2*	*VHL*
*WRN*	*WT1*	*XPA*	*XPC*						

NGS, next-generation sequencing.

**Table 4:** Panel of 26 genes used for NGS analysis through SOPHiA Hereditary Cancer Solution (HCS).

*ABRAXAS1*	*APC*	*ATM*	*BARD1*	*BRCA1*	*BRCA2*	*BRIP1*,	*CDH1*	*CHEK2*,	*EPCAM*
*MLH1*	*MRE11*	*MSH2*	*MSH6*	*MUTYH*	*NBN*	*PALB2*	*PIK3CA*,	*PMS2*,	*PTEN*,
*PIK3CA*	*RAD50*	*RAD51C*	*RAD51D*	*STK11*	*TP53*	*XRCC2*			

NGS, next-generation sequencing.

### Variant Classification

Genetic variants identified were classified according to the International Agency for Research on Cancer (IARC) recommendations (8), and pathogenic (PV; class 5) and likely pathogenic (LPV; class 4) were taken into consideration. The classification of *BRCA1/2* and *FLCN* variants was performed through the main mutation databases and tool prediction software—BRCA Shar (formerly Universal Mutation Database), Leiden Open Variation Database (LOVD), BRCA Exchange, ClinVar, dbSNP, HCI Cancer Susceptibility Genes Prior Probabilities of Pathogenicity, and Varsome (9)—and were categorized according to the available clinical interpretation (10). Variants automatically annotated by the platform were manually checked on the main human genomic databases.

## Results

**Figure 1:**
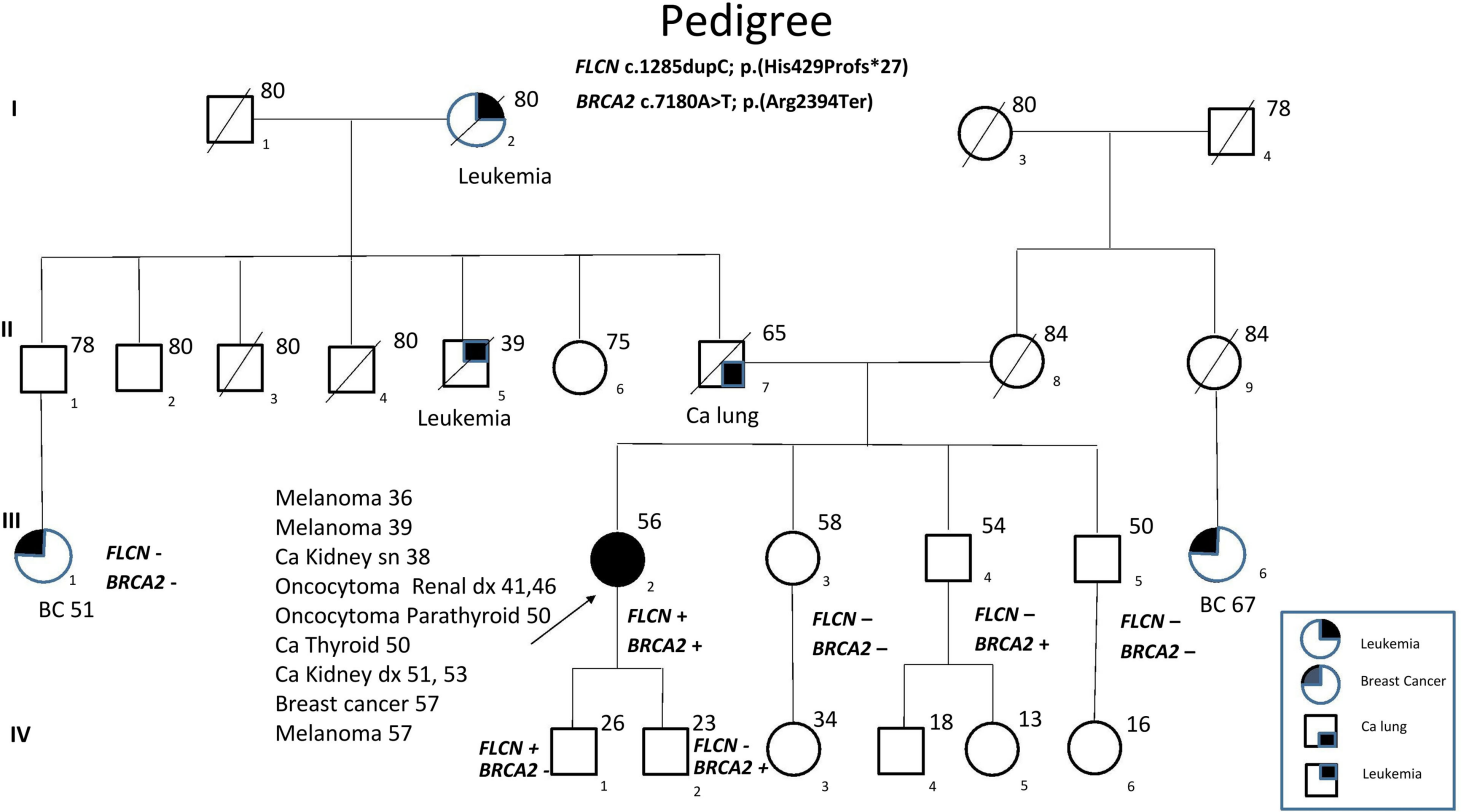
Pedigree of the family with disease-associated *FLCN/BRCA2* mutations. Circles represent females, and squares represent males. Symbols with a quarter represent cancer patients. Symbols with a slash indicate deceased individuals. The arrow points to the proband.

*FLCN* variant c.1285dupC; p.His429Profs*27, a frameshift mutation in heterozygosity predicted to be a disease-causing mutation, was detected in the proband patient and further observed in one family member (**Table 5**), confirming the BHDS diagnosis. After confirmation of the diagnosis, the whole family underwent a specific analysis program. None of the two brothers and sister was found to be carriers of the same pathogenic variant in *FLCN*, so they were assigned an equal population cancer risk and were monitored periodically, in accordance with the screening program. *FLCN* gene was found to be normally expressed in the younger son, while the oldest one was found to be a carrier. For this reason, the oldest son was subjected to a very careful and closely monitored screening program. Furthermore, as an incidental finding, the MGP analysis highlighted the presence of a pathogenic variant also in *BRCA2* gene in 3 out of 7 members of the family (**Table 5**). For that reason, other than the indicated screening of BHDS, we recommended close screening for increased risk of breast and ovarian cancers, according to the high-risk program. More in detail, the identified variant *BRCA2* referred to the mutation c.7180A>T; p.(Arg2394Ter), and to our knowledge, no other cases were observed so far to be carrying mutations in both *FLCN* and *BRCA2* genes. The details about pathogenic *FLCN* and *BRCA2* variants detected are listed in **Table 6**. The pedigree of family members harboring pathogenic *FLCN* and *BRCA2* variants is shown in **Figure 1**. Insights on the *BRCA* analysis performed on relatives pointed out one of two brothers as the carrier of the same pathogenic variant in *BRCA2*. He was therefore subjected to periodic controls, particularly about prostate and breast. In addition, the younger proband’s son was found to be a *BRCA2* mutation carrier. The two proband’s nephews, and the son and daughter of *BRCA2* mutation carrier brother are actually too young to undergo genetic analysis to identify their eventual carrier status. Later, a first paternal cousin developed breast cancer (BC) at 50 years old but have wild-type *BRCA2* and *FLCN*. It was not possible to perform the same analysis for the parents, as they have died, so it cannot be established whether the origin of the two mutations was paternal or maternal. Furthermore, a maternal cousin who experienced BC at 67 years old declined to undergo the analysis.

**Table 5:** *FLCN* and *BRCA2* variants identified in the proband and in family members.

Patients (ID sample and relative grade)	*FLCN* variant	*BRCA2* variant
E07/01 (III2)	c.1285dupC; p.His429Profs*27	c.7180A>T; p.(Arg2394Ter)
E07/02 (III4)	wt	c.7180A>T; p.(Arg2394Ter)
E07/03	wt	wt
E07/04	wt	wt
E07/05	wt	wt
E07/06 (IV1)	c.1285dupC; p.His429Profs*27	wt
E07/07 (IV2)	wt	c.7180A>T; p.(Arg2394Ter)

**Table 6:** List and details of pathogenic FLCN and BRCA2 variants detected in the family.

Gene	Genome position	c.DNA change	Protein change	Variant type	Consequence	Exon rank	VAF	gnomAD allele frequency	IARC class	dbSNP/ClinVar
*FLCN*, NM_144997	Chr17: 17119708	c.1285dupC	p.His429Profs*27	Indel	Frameshift	11	43.9%	3.95e−5	C5	rs80338682/pathogenic
*BRCA2*, NM_000059	Chr13: 32929170	c.7180A>T	p.Arg2394*	SNP	Nonsense	14	45.8%	6.57e−6	C5	rs80358946/pathogenic

gnomAD allele frequency refers to v3.1.2. dbSNP/ClinVar mutation classification according to the Single Nucleotide Polymorphism database (dbSNP) and Clinical Variant (ClinVar).

VAF, variant allele frequency; IARC, International Agency for Research on Cancer.

## Discussion

BHDS is a rare hereditary syndrome with high exposure to recurrent PNX correlated to multiple lung cysts and an increased risk of experiencing renal cancer and fibrofolliculomas in the skin. Multiple lung cysts are observed in approximately 67%–90% of patients with BHDS; and in about 40% of subjects, the onset of PNX is typical (11). Furthermore, a notably considerable proportion of BHDS families developed colorectal cancer (CRC) before the age of 50 years, or more than two family members are affected by CRC (12). Management of BHDS depends upon the clinical manifestation of the phenotype. It is extremely important to identify this rare syndrome at early stages, both for patients and also for subjects with a positive family history of RCC and PNX. *FLCN* sequencing should be taken into account in patients and their families since the incidence of renal cancer in BHD patients is very high, and detection at early stages can prevent its metastasis. Interestingly, it has been suggested that certain *FLCN* variants lead to a form of BHDS with PNXs but no renal carcinomas, although the risk of developing fatal renal cancers with crucial consequences is high (13). In this report, we present the case of a patient who was diagnosed with BHD syndrome after the manifestation of multiple serious symptoms and in whom a c.1285dupC; p.His429Profs*27 mutation on exon 11 of *FLCN* gene was identified. This variant is among the most frequent BHD mutations, and exon 11 represents the most common mutation site for this rare pathology (14). At once, we could not think of a disorder consistent with hereditary breast and ovarian cancer (HBOC), as the patient had neither breast nor ovarian tumors, and BC cases in paternal and maternal lines were not sufficient to consider a hereditary–familial form. The symptoms presented by the patient (PNX and oncocytoma) were suspicious for BHDS. Curiously, as an incidental finding, we also detected a c.7180A>T; p.(Arg2394Ter) nonsense mutation on exon 14 of *BRCA2* gene, a type of variant already identified in families at risk for HBOC (15), but without previous correlations with BHDS. The diagnosis of BHDS, carried out primarily thanks to the genetic counseling service and the confirmation of *FLCN* mutation, was able to guarantee life-extending treatments for the patient and surely an improved long-term survival. To date, the patient is currently in stable condition, on dialysis, and under letrozole treatment for BC and nivolumab for kidney cancer metastasis. Certainly, in most cases, prognosis depends upon the occurrence of histologic type of renal cancer, and the majority of deaths results from metastatic diseases due to clear cell carcinoma (16). Subsequently, of extreme importance, it was possible to allow family members to undergo a surveillance screening program for the possible detection of the same genetic disorder or correlated symptoms. A periodic screening was recommended to family members, and it is noteworthy that three individuals were identified to be carriers of *FLCN* or *BRCA2* mutations through family tracing; therefore, they are more likely not to develop complications, as they are being strictly monitored. This case supports the importance of early diagnosis of the syndrome and highlights the relevance of MGPs in identifying patients with the co-existence of pathogenic germline mutations that could be unfavorable for the risk of cancer development. In our case report, we describe how these analyses allowed unaffected carriers to have a guaranteed specific surveillance strategy. Although it was not possible to recover DNA from all family members, especially from the proband’s parents, the genetic analysis of *FLCN* and *BRCA2* genes involved in hereditary cancers, combined with the family history, ensured to outline a more accurate diagnosis and to assess the risk of specific cancers of the family. The MGP represents an important tool to gain insight into the mechanisms that lead to high susceptibility to certain tumors and the interactions between causative mutations. Moreover, background mutations, as for *BRCA* in this family, increase the knowledge of the connections between the genotype and the phenotype of the family. Overall, the findings of the case we presented, combined with the *BRCA* variant identified as the incidental report, have enhanced our understanding of BHDS, raising the surveillance threshold for this syndrome also for any other future cases and allowing strict monitoring programs for family members involved, which could ensure long-term benefits.

## Data Availability Statement

The datasets presented in this article are not readily available because they are part of genetic data obtained from analyzes of patients. Requests to access the datasets should be directed to corresponding author erika.bandini@irst.emr.it.

## Ethics Statement

The studies involving human participants were reviewed and approved by Ethics Committee IRST IRCCS-AVR, 2207/2012. The patients/participants provided their written informed consent to participate in this study.

## Author Contributions

VZ and RD conceived the study. IC, VAr, VAn, GP, LP, and VZ performed the experiments and analyzed and interpreted the data. VAr, MR, GM, FF, DC, and RD were responsible for clinical resources and management. EB wrote the manuscript. RD was responsible for the supervision of the project. All authors contributed to the article and approved the submitted version.

## Conflict of Interest

The authors declare that the research was conducted in the absence of any commercial or financial relationships that could be construed as a potential conflict of interest.

## Publisher’s Note

All claims expressed in this article are solely those of the authors and do not necessarily represent those of their affiliated organizations, or those of the publisher, the editors and the reviewers. Any product that may be evaluated in this article, or claim that may be made by its manufacturer, is not guaranteed or endorsed by the publisher.
